# Supplementary material for: Chromothripsis during telomere crisis is independent of NHEJ, and consistent with a replicative origin
Source: Genome Res. 2019 May;29(5):737–49. doi: 10.1101/gr.240705.118 (PMC6499312; doi:10.1101/gr.240705.118)
Supplement: Supplemental Material [file supp_gr.240705.118_Supplemental_file_1.zip › contigs/annotated_contigs/DB79/contig.2.DB79_length_311_mean_cov_4.34083601286.docx]

**DB79_length_311_mean_cov_4.34083601286**

```
AAAGGAGTGCTTGAATTTGACTTTAAAAACTGGAGTACCATAGTGCAGTGCTT|AA|CAATGGATTTCTGGATCCCTCCTCATTGCTTT
 >chr6:140030970-140031025 + E=1e-21                  >chr6:140035088-140035346 - E=2e-
TGAATCAAAATTTCTGGGAATGCATTCCAAGAATTTGAATATCATCCAACATCTGGGCAATTCTTACATTCAGTAAGTCTGAGTATCTC
144
TGCCCTAAGCCAAGCACATGGTTAGCCACAGTAACAGTGGCTCACACATTTACTGATCTTTGAGACCATCAGGAATTTTCAAACATCAG

TGTGCCTAAAGAGAAAAAAACACGATTCACGTCTTAAATACGAAAA
```
